# Behaviour change techniques and contraceptive use in low and middle income countries: a review

**DOI:** 10.1186/s12978-015-0091-y

**Published:** 2015-10-30

**Authors:** Mwelwa Phiri, R. King, J. N. Newell

**Affiliations:** Public Health Practitioner, Lusaka Trust Hospital, 2191, Nsumbu road, P.O .Box 35852, Lusaka, Zambia; Nuffield Centre for International Health and Development, Leeds Institute of Health Sciences, Clarendon Road, Leeds, LS2 9LJ UK

**Keywords:** Contraception, Community-based intervention, Behaviour change techniques, Low and middle income country

## Abstract

We aimed to identify effective behaviour change techniques to increase modern contraceptive use in low and middle income countries (LMICs). Literature was identified in Global Health, Web of Science, MEDLINE, PsycINFO and Popline, as well as peer reviewed journals. Articles were included if they were written in English, had an outcome evaluation of contraceptive use, modern contraceptive use, contraceptive initiation/uptake, contraceptive adherence or continuation of contraception, were a systematic review or randomised controlled trial, and were conducted in a low or middle income country. We assessed the behaviour change techniques used in each intervention and included a new category of male partner involvement. We identified six studies meeting the inclusion criteria. The most effective interventions were those that involve male partner involvement in the decision to initiate contraceptive use. The findings also suggest that providing access to contraceptives in the community promotes their use. The interventions that had positive effects on contraceptive use used a combination of behaviour change techniques. Performance techniques were not used in any of the interventions. The use of social support techniques, which are meant to improve wider social acceptability, did not appear except in two of the interventions. Our findings suggest that when information and contraceptives are provided, contraceptive use improves. Recommendations include reporting of behaviour change studies to include more details of the intervention and techniques employed. There is also a need for further research to understand which techniques are especially effective.

## Introduction

More than 80 % of unintended pregnancies occur in low and middle income countries (LMICs). With an unmet need for contraceptives of about 40 %, millions of women lack access to contraceptives [1, 2]. As a result, unsafe abortion is highly prevalent and contributes to high maternal mortality rates in LMICs [3]. More than 90 % of abortion related mortality and morbidity can be avoided by using contraceptives [1, 3]. In LMICs, contraceptive use also has a significant impact on child mortality, as well as having economic benefits [3, 4].

Community-based interventions to increase contraceptive use have been implemented in LMICs and have proved useful in reaching rural, hard to reach populations with limited access to static service delivery of contraceptives [3, 5]. However, the cost involved in increasing the reach of interventions is a hindrance. Cost, coupled with an uncertainty of what really works in changing reproductive behaviour [3, 5], is not conducive to scaling up interventions.

Most interventions, while having a component of behaviour change, are not clear about the behaviour change techniques (BCTs) employed [6, 7]. BCTs are the essential building blocks of interventions [7]. It is important that health practitioners learn these techniques, as this allows community interventions to be replicated successfully. Where details of BCTs are not shared, this can lead to interventions being compromised [7]. It is therefore important to identify what techniques work to change reproductive behaviour in LMICs, as this will ensure programmes are designed effectively and that scarce resources are used appropriately and interventions are cost-effective [7].

This review identifies effective community-based interventions that increase modern contraceptive use in LMICs, somewhat akin to existing reviews [5, 8], however, our primary aim is to identify specific BCTs that have been proved to be effective in randomised controlled trials (RCTs), and provide recommendations on which particular techniques should be included in the design of future interventions.

For the purposes of this review, contraception refers to use of modern contraceptives including:

- long acting methods such as male and female sterilisation, IUDs, implants, and injectables; and
- Short acting methods such as pills, male and female condoms and other supply methods, such as spermicides.

However, we acknowledge that contraception does not merely encompass modern contraceptives [9].

## Methods

### Search strategy

The following databases were used to identify relevant articles: Global Health, Web of Science, MEDLINE, PsycINFO and Popline. Peer reviewed journals searched were Social Science and Medicine, Contraception, Studies in Family Planning, Reproductive Health Matters and Health Education Research.

Key search terms were “low and middle income countries”; “developing countries”; “contraception”; “family planning”; “randomised controlled trials”; and “systematic reviews”. Subject headings of the key terms and text word terms were used for a comprehensive search. Reference lists of identified articles were also searched. The search was limited by the type of study design (Systematic reviews and randomised controlled trials) and the above search terms. This restriction to RCTs and Systematic reviews is because they are the two study designs which will answer our research question particularly on the effectiveness of interventions and robustness of the interventions. The studies were assessed on relevance to the topic. This is because of the varied meanings of community-based interventions. This broad search strategy was developed with Dr Rebecca King and Judy Wright (Information specialist). Summary journal searches are in Table 1.

**Table 1:** Summary of journal search strategyᅟ

Journal			No. of hits	Search terms
Social science and medicine			744	(family planning OR contraceptive use AND developing countries OR low and middle income countries OR low income countries OR third world countries)
Contraception			664	Keywords : family planning OR contraceptive use in developing countries
Studies in family planning			525	family planning OR contracept* AND developing countries in Studies in Family Planning
			411	((family planning OR contracept* AND developing countries OR low AND middle income countries OR low income countries OR third world countries AND systematic review* OR randomized controlled trial*) AND jid:(j100383)) AND (systematic reviews OR RCTs OR interventions)
Reproductive health matters			882	Used keywords of family planning and contraceptive use
Health education research			267	Searching journal content for family planning, contracept* (any words) in title or abstract and family planning, contraception, developing countries, reviews, randomized controlled trials (any words) in full text.

### Screening and papers selection criteria

Two researchers screened titles and abstracts independently and discussed any differences and areas for further assessment. Full text assessments were done by the primary author and the shortlisted articles were independently reviewed by the second author for inclusion, differences were resolved by discussion and consultation with the third author.

Any differences in assessment were resolved by a third researcher.

Our inclusion criteria were:

- English language papers;
- Studies with an outcome evaluation of contraceptive use, modern contraceptive use, contraceptive initiation/uptake, contraceptive adherence or continuation of contraception;
- Systematic reviews and RCTs in LMICs (World Bank classification); and
- Studies meeting methodological rigour of behaviour change intervention assessment based on four criteria by Michie and Abraham [10]. These criteria are: 1) random allocation or matched control group; 2) pre and post intervention data reporting; 3) reporting intention to treat analysis; and 4) reporting all outcomes indicated by aims and objectives of the study [10].

### Assessing behaviour change techniques

Authors were contacted for additional information about the BCTs they used, or for further descriptions of the methodologies they used. To identify BCTs, we used the method identified by Briscoe and Aboud [6] who classified 26 BCTs initially identified by Abraham and Michie [7] into six broad categories which included new techniques not included in the Abraham and Michie [7] list. These categories were: 1) information; 2) performance; 3) problem solving; 4) social support; 5) materials; and 6) media. We also identified a new category: 7) male partner involvement.

## Results

### Search flow

**Fig. 1:**
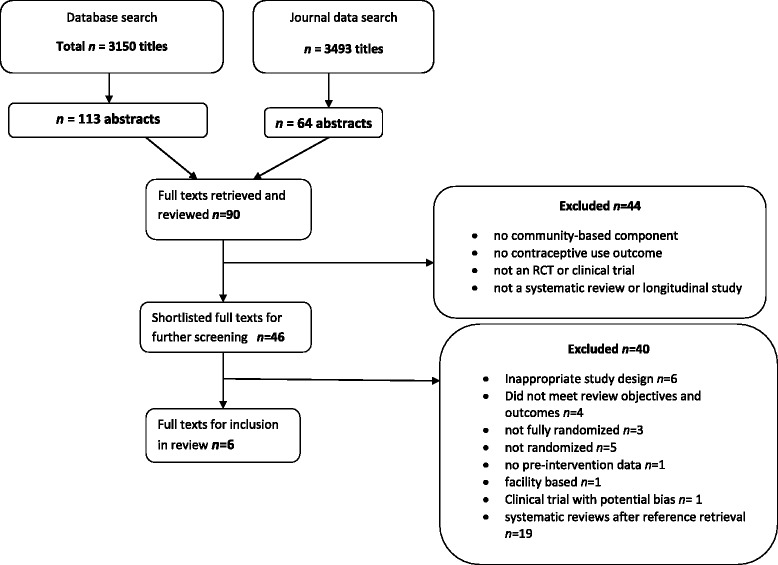
Data Evaluation Flow Chart

The reviewers identified 3150 publications in the data base search and 3493 publications in the journal search. After screening titles and abstracts and removing duplicates, 90 studies were selected for further assessment. After further screening, six studies were included in the review. Details are provided in Fig. 1.

### Study details

The studies reviewed were published in 5 journals. These were conducted in Syria, Ethiopia, Zambia, Malawi, Nepal and Uganda. All studies were RCTs. Table 2 gives a brief description of the studies included in the review.

**Table 2:** Summary of interventions

Authors	Country conducted in	Delivered by and where	Recipient of intervention	Specific contraceptives included	Duration and intensity	Design of intervention	Post test and follow-up	Sample size
Bashour et al. 2008	Syria	Registered midwives: Home Visit (HV)	Post-partum women	Pills, IUD, condoms, natural methods.	5 home visits on day 1, 3, 7 and 30.	RCT	4 months postpartum	876 women
Terefe and Larson 1993	Ethiopia	Trained TBA and female HA (HV)	Married women living with husbands	Pills, copper IUD and condoms	2 home visits	RCT	2 months and 12 months post intervention	527 couples
Stephenson et al. 2011	Zambia	Video and counsellor	HIV-serodiscordant and concordant couple	Condoms, pills, injectables, Norplant, IUD and tubal litigation	30 min video and Q&A with counsellor	RCT	immediately	1178 couples (condoms and nonusers)
Shattuck et al. 2011	Malawi	Male motivator(HV)	Male partners	Condoms, injectables, pills, IUD, diaphragms and male sterilization	5 visits over 6 months	RCT	7 months. 1 month after last visit.	400 men
Bolam et al. 1998	Nepal	Female health educators, midwives & CHW(HV)	Post-partum women	Not detailed.	1 post-partum, 1 home visit	RCT	6 months	540 women
Lutalo et al. 2010	Uganda	SDPs, COBRAs, opinion leaders, users, TBAs(HV)	Sexually active non pregnant women, sexuallyactive men	Pill, injectables and condoms	3 years continuous activity	RCT	3 years post intervention	10,294 couples

### Key findings

The most effective interventions appear to be those that involve male partner involvement in the decision to initiate contraceptive use [11–14]. This is shown in Table 3. However, two of these studies [11, 13] consisted of self-reporting of contraceptive use either in the presence of the male partners or by the male partners. As the methods included in the studies were female user dependent (pills and injectables), this could affect the validity of the results, as male partners may not have been present when their female partners used the contraceptive.

The studies conducted among post-partum women suggest that interventions targeting women only are not as effective as those that target couples. In the Bashour et al. [15] study using educative post-natal home visits by midwives in Syria, there was no significant difference in contraceptive uptake between research arms for contraceptive use (42 %, 37 % and 40.5 %). This low evidence of effectiveness is congruent with a Cochrane review on post-natal education, which found low evidence of effectiveness and suggested further research [8].

The findings also suggest that providing immediate access to contraceptives in the community promotes their use. Trials of interventions that included onsite provision, as opposed to referral to health facilities, [12–14] showed better uptake than the other studies that did not provide the contraceptives. However, the uptake in all the studies were fairly modest. While the study by Stephenson et al on serodiscordant couples appeared to be effective, it was conducted among HIV positive serodiscordant and concordant couples. This could affect how the intervention is used with other target groups, as the HIV status of the couples could be a major determinant in whether they use family planning [14].

### Behaviour change techniques

Table 3 identifies which interventions utilised which techniques and the effectivness of the interventions. We identified BCTs from four of the six Briscoe and Aboud [6] categories. We also identified male partner involvement as an additional category that had not been explicitly identified. The most successful interventions used multiple BCTs as shown in Table 3.

**Table 3:** Summary of behaviour change techniques using Briscoe and Aboud’s [7] categories

Study	Outcomes	Effectiveness	Observed behaviour	Techniques of behaviour change						
				Information	Performance	Problem solving	Social support	Materials	Media	Male partner involvement/participation
Bashour et al. 2008	Contraceptive uptake	Intervention- 37 %;40 % and 42 % across arms	No significant difference between arms. Self-reported	Health education	-	Discussion of problems	-	-	-	-
Terefe and Larson 1993	Contraceptive use	Intervention 47 %,control 33 %	Contraceptive use and continuation	Health education	-	-	-	If requested, pills and condoms	-	Couple administered information
Stephenson et al. 2011	Modern contraceptive uptake and switching	baseline use 21.5 %, after intervention-93.6 %	Uptake of contraceptives, switching and addition if using condoms only	Information	-	-	-	If requested, pills, injectables, Norplant	Video, Print media	Couple administered intervention
Male motivator project, Shattuck et al. 2011	Family planning uptake	intervention-78 %, control-59 %	Self-reported	Information from peers	-	-	Peers	-	-	Male partner only administered intervention
Bolam et al. 1998 postnatal health education	Uptake of FP services or contraceptive use, Two different outcomes, self-reported	intervention-20 %, control-14 %	Self-reported	Information on importance of FP, location of nearest clinic, choice of methods.	-	-	-	-	Cloth flip charts developed by local artist.	-
Lutalo et al. 2010	Contraceptive use prevalence	intervention- 23 %, control-20 %	Contraceptive use and uptake	Information at meetings.	-	-	Opinion leaders and certified users of FP, volunteers chosen by communities	Provision of pills, condoms	Videos, role play, drama, IEC material- leaflets, booklets and posters	-

#### Technique one: information techniques

Information techniques were used in all the interventions and included providing information on the available methods, importance and advantages of methods, ill effects of large families, misconceptions about methods and explanations of methods, including side effects and efficacy [11–16]. This information was mostly provided orally and face-to-face. Two interventions used a video to provide information with one adding a description of the methods on the patient consent form. One intervention also used drama, role playing and music to provide information. Peers, authority figures such as healthcare professionals, and community health workers (CHWs) provided this information.

#### Technique two: performance techniques

None of the interventions included modelling behaviour, observation, demonstrations or practice by the participants. One intervention included providing instructions for correct condom use [11] but did not explicitly perform or demonstrate correct condom use.

#### Technique three: problem-solving techniques

The technique of ‘discussing any problems and help’ during a home visit was used and described in detail in one of the interventions [15]. However, this was not explicitly used for contraceptive uptake. Problem-solving techniques were mentioned in one other intervention but were not adequately described.

#### Technique four: social support techniques

Social support techniques included using male role models (or peers) to normalise contraceptive use, training opinion leaders to disseminate family planning information, involving community residents to choose volunteer role models from within the community, and using authority figures such as health practitioners. Social support techniques were used in two interventions.

#### Technique five: providing materials

Providing materials included providing contraceptive pills and condoms on site. Injectables were provided only in the event of a medical practitioner volunteer being present in one of the interventions. Social marketing was used in one of the interventions for providing the pill, injectables and condoms. Material provision was used by three of the six interventions. If contraceptives could not be provided, participants were given information on where to access them.

#### Technique six: media techniques

Media techniques consisted of using short videos to provide information, illustrating scenarios using drama and role plays, music and print media such as flip charts, leaflets, booklets and posters. Print media was provided as a supplement to the oral information in two of the interventions. None of the interventions used mass media. One intervention also contained a brief description of the methods on the patient consent form. Media techniques were used by three of the six interventions.

#### New technique: male partner involvement

Involving male partners is a technique that was used in three of the interventions. In two of the interventions this was by delivering the intervention in the presence of either the husband or partner with the female partner. Methods used included providing information and contraceptives. One intervention used information techniques that specifically targeted the male partners only.

## Discussion

The results described above of the included studies suggest several things, firstly, that the most effective interventions appear to be those that involve male partner involvement in the decision to initiate contraceptive use and utilise a combination of behaviour change techniques as opposed to use of only one technique [11-14]. This is also why the authors identified this as a new and separate category. However, two of the studies [11, 13] consisted of self reporting of contraceptive use either in the presence of the male partners or by the male partners, and as the methods included in the studies were female user dependent (Pills and injectables), this could be a possible source of bias and could potentially affect the validity of the results.

Secondly, the results also suggest that interventions that provide access to contraceptives on site at the time of implementation promote use. This is alluded to and is suggested as a prompt to use in the studies in Zambia, Ethiopia and Uganda as opposed to referral to health facilities, this is supported as the results in these countries are better than for the other reported studies [12–14]. However, the results in all the studies were fairly modest and did see an increase in use across all arms. It should be noted that while the study in Zambia appeared to be effective, it was conducted among HIV positive serodiscordant and concordant couples, this could affect the applicability of the intervention in other target groups and the HIV status of the couples could be a major determinant in the use of contraceptives [14].

It should also be noted that all the interventions consisted of married or cohabiting couples, which would also impact the applicability of the interventions to groups such as adolescents or unmarried women and other underserved populations.

Thirdly, the results of studies conducted among post partum women only suggest that interventions that target women only are not as effective as those that target couples. In the study conducted in Syria, there was no difference between arms in terms of contraceptive use and there was only a small albeit significant effect in the study conducted in Nepal [15, 16]. These results however should be taken with caution considering these two trials used only one or two BCTs. This low evidence of effectiveness is however, congruent with a Cochrane review on post natal education which found the evidence of effectiveness as low and suggested further research [8].

These results also suggest that interventions that include home visits do have more of an impact and are a component of all but one of the interventions.

### Limitations of this review

One of the limitations of this review was that it was restricted to English language only papers; this could have resulted in missed studies published in languages other than English. Another limitation is it was restricted to RCTs only and so could have missed studies that could have been effective and had used other BCTs that were not identified in our review. Also, based on our definition of CBI, this could have limited our results. However, the review used general search terms for contraception use and authors were contacted for additional information particularly with regard to behaviour change techniques or for any further description of the methodology and the techniques. Additionally, most of the interventions were not reported in detail and therefore it was difficult to extract information on the BCTs. Finally, as all the interventions targeted married or cohabiting couples, the BCTs may not be applicable to underserved populations such as adolescents and unmarried women.

### Recommendations

The findings described and discussed above provide some insight into what could work to increase family planning use in LMIC. Despite having reporting positive results, the findings would require further research and evaluation to be able to generalise the findings. Further projects and research could include male involvement as it has shown to be promising, however, the context in which the intervention is taking place should be considered particularly with regard to gender and power in that particular context. The design of the further research recommended should be stronger to better illustrate effectiveness.

We would also recommend the inclusion of more details in reporting, particularly with regard to BCTs to allow for reliability of what works and also for disaggregation of the effect of individual BCTs. The costs involved may be limiting in this instance but the detailed reporting would be very useful for future implementation and replication.

We would also recommend the use of multiple BCTs to increase contraceptive use, with at least provision of information about the methods and the provision of contraceptives on site as opposed to referrals to static health facilities as the studies which provided on site materials showed greater strength. BCT categories that support the first two categories and could be included are the use of media, social support and role models.

Finally, as it is difficult to disaggregate the effectiveness of individual techniques, we recommend further research to understand which techniques are especially effective. In particular, we recommend further investigation of performance techniques, problem-solving techniques and social support techniques. However, it may be the case that only a combination of BCTs will be effective.

## Abbreviations

BCT: Behaviour change technique
CHW: Community health worker
LMIC: Low and middle income country
RCT: Randomised controlled trial
HV: Home visit
